# Perspectives and Emotional Experiences of Patients With Chronic Myeloid Leukemia During ENESTPath Clinical Trial and Treatment-Free Remission: Rationale and Protocol of the Italian Substudy

**DOI:** 10.3389/fonc.2021.638689

**Published:** 2021-05-26

**Authors:** Lidia Borghi, Gianantonio Rosti, Alessandro Maggi, Massimo Breccia, Eros Di Bona, Alessandra Iurlo, Gaetano La Barba, Paolo Sportoletti, Francesco Albano, Sara Galimberti, Flavia Rivellini, Giovanna Rege Cambrin, Isabella Capodanno, Antonio Cuneo, Massimiliano Bonifacio, Simona Sica, Luca Arcaini, Enrico Capochiani, Claudia Minotto, Fabio Ciceri, Monica Crugnola, Luigi Di Caprio, Sharon Supekar, Chiara Elena, Michele Baccarani, Elena Vegni

**Affiliations:** ^1^ Department of Health Sciences, University of Milan, Milan, Italy; ^2^ Department of Hematology-Oncology, L. and A. Seràgnoli, University of Bologna, S. Orsola-Malpighi Hospital, Bologna, Italy; ^3^ Hematology Division, Hospital S.G. Moscati, Taranto, Italy; ^4^ Department of Translational and Precision Medicine, University Sapienza Rome - Azienda Policlinico Umberto I, Rome, Italy; ^5^ Department of Hematology, Vicenza Hospital, Vicenza, Italy; ^6^ Hematology Division, Foundation IRCCS Ca’ Granda Ospedale Maggiore Policlinico, Milan, Italy; ^7^ Department of Hematology, Spirito Santo Hospital, Pescara, Italy; ^8^ Institute of Hematology-Centro di Ricerche Emato-Oncologiche, Department of Medicine, University of Perugia, Perugia, Italy; ^9^ Department of Emergency and Organ Transplantation, Hematology Section, University of Bari, Bari, Italy; ^10^ Department of Clinical and Experimental Medicine, Section of Hematology, University of Pisa, Pisa, Italy; ^11^ Hematology Unit, Nocera Inferiore Hospital, Nocera Inferiore, Italy; ^12^ Division of Hematology and Internal Medicine, San Luigi Gonzaga University Hospital, Orbassano, University of Turin, Turin, Italy; ^13^ Division of Hematology, AUSL-IRCCS Reggio Emilia, Reggio Emilia, Italy; ^14^ Institute of Hematology, University of Ferrara, Ferrara, Italy; ^15^ Department of Medicine, Section of Hematology, University of Verona, Verona, Italy; ^16^ Fondazione Policlinico Universitario Agostino Gemelli – IRCSS, Università Cattolica del Sacro Cuore, Rome, Italy; ^17^ Department of Hematology Oncology, IRCCS S. Matteo Hospital Foundation, Pavia, Italy; ^18^ Department of Oncology, Azienda Toscana Nord Ovest, Livorno, Italy; ^19^ Department of Oncology and Hematology, Aulss 3 Serenissima, Venice, Italy; ^20^ Hematology and Bone Marrow Transplantation Unit, IRCCS San Raffaele Scientific Institute, Milano, Italy; ^21^ Division of Hematology and BMT Center AOU Parma, Parma, Italy; ^22^ Oncology Region Europe, Novartis Farma SpA, Origgio, Italy; ^23^ Department of Hematology and Oncology “L. and A. Seràgnoli”, University of Bologna, Bologna, Italy

**Keywords:** Chronic myeloid leukemia, ENESTPath, emotional experience, nilotinib, psychological distress, quality of life, study protocol, mixed methods

## Abstract

Achievement of deep molecular response following treatment with a tyrosine kinase inhibitor (TKI) allows for treatment-free remission (TFR) in many patients with chronic myeloid leukemia (CML). Successful TFR is defined as the achievement of a sustained molecular response after cessation of ongoing TKI therapy. The phase 3 ENESTPath study was designed to determine the required optimal duration of consolidation treatment with the second-generation TKI, nilotinib 300 mg twice-daily, to remain in successful TFR without relapse after entering TFR for 12 months. The purpose of this Italian ‘patient’s voice CML’ substudy was to evaluate patients’ psycho-emotional characteristics and quality of life through their experiences of stopping treatment with nilotinib and entering TFR. The purpose of the present contribution is to early present the study protocol of an ongoing study to the scientific community, in order to describe the study rationale and to extensively present the study methodology. Patients aged ≥18 years with a confirmed diagnosis of Philadelphia chromosome positive *BCR-ABL1*+ CML in chronic phase and treated with front-line imatinib for a minimum of 24 months from the enrollment were eligible. Patients consenting to participate the substudy will have quality of life questionnaires and in-depth qualitative interviews conducted. The substudy will include both qualitative and quantitative design aspects to evaluate the psychological outcomes as assessed *via* patients’ emotional experience during and after stopping nilotinib therapy. Randomization is hypothesized to be a timepoint of higher psychological alert or distress when compared to consolidation and additionally any improvement in health-related quality of life (HRQoL) due to nilotinib treatment is expected across the timepoints (from consolidation, to randomization, and TFR). An association is also expected between dysfunctional coping strategies, such as detachments and certain personality traits, and psychological distress and HRQoL impairments. Better HRQoL outcomes are expected in TFR compared to the end of consolidation. This substudy is designed for in-depth assessment of all potential psycho-emotional variables and aims to determine the need for personalized patient care and counselling, and also guide clinicians to consider the psychological well-being of patients who are considering treatment termination.

NCT number: NCT01743989, EudraCT number: 2012-005124-15

## Introduction

Chronic myeloid leukemia (CML) is a hematopoietic disorder, characterized by the rearrangement of the fusion oncogene *BCR-ABL1* that encodes an oncoprotein with dysregulated ABL1 tyrosine kinase activity (1–3). The advent of tyrosine kinase inhibitors (TKIs) that target BCR-ABL1 has dramatically changed the Philadelphia chromosome-positive (Ph+) CML therapeutic landscape (4). The first-generation TKI, imatinib, has shown high clinical response rates and long-term survival in the vast majority of patients (90%) in Ph+ CML chronic phase (CML-CP) (5). Front-line use of the second-generation TKI, nilotinib, has been associated with the achievement of a rapid and deep molecular response (DMR) in a large proportion of patients (88%), where a DMR is defined as a molecular response greater than a 4-log reduction in *BCR-ABL1* transcripts (≥MR^4.0^) on the International Scale (IS) (6). With the achievement of a sustained DMR in a significant number of patients treated with TKIs, treatment-free remission (TFR) has become a new treatment goal in CML (7, 8).

Treatment-free remission is usually attained with undetectable minimal residual disease or minimal residual disease detectable at a stable low level, without the need for ongoing treatment (8). Several studies have shown that the achievement of early and sustained deeper response is associated with successful TFR after treatment cessation (9–17). The observational STOP 2G-TKI study included patients with undetectable *BCR-ABL1* for at least 2 years on front-line or second-line treatment with nilotinib or dasatinib (10).

Of the Evaluating Nilotinib Efficacy and Safety in Clinical Trials (ENEST) - TFR studies, the ENESTfreedom trial was designed to evaluate the potential of TFR in patients with MR^4.5^ on front-line nilotinib (6), while ENESTgoal, ENESTop and ENESTPath were conducted in distinct populations of patients treated with second-line nilotinib (18–20). A few nilotinib discontinuation studies have reported successful TFR in 50% to 68% of patients, without molecular relapse or recurrence after 1 to 2 years (15, 21–23). Data from the phase 3 ENESTcmr trial support switching to nilotinib in patients with persistent minimal residual disease following long-term imatinib use (24). The largest prospective TFR trial, the EUROpean Stop tyrosine Kinase Inhibitor (EURO-SKI) study, was conducted in patients with sustained DMR on imatinib, nilotinib or dasatinib (25). The patient eligibility criteria for stopping TKIs and attempting TFR in clinical trials are described in the National Comprehensive Cancer Network (NCCN) and European LeukemiaNet guidelines (7, 26).

The TFR study, ENESTPath, is a prospective, randomized, open-label, two-arm (nilotinib 300 mg twice-daily [BID] for 2 years or 3 years) ongoing phase 3 study to evaluate the TFR rate in patients with CML-CP achieving sustained DMR after 2 to 3 years of second-line nilotinib treatment. This active study enrolled 620 patients with CML-CP from 22 European countries between May 2013 and April 2015. The primary objective of the ENESTPath trial was to determine the optimal duration of nilotinib consolidation treatment (12 months versus 24 months) for patients to remain in TFR (≥ MR^4.0^) without molecular relapse for 12 months upon treatment discontinuation and after entering the TFR phase (18). The interim data of the ENESTPath trial support switching to nilotinib 300 mg BID from imatinib, with higher rates of MR^4^ and MR^4.5^, thereby increasing the probability of achieving TFR (18).

In addition to molecular response, psychological and emotional factors are considered to have an impact on a patient’s decisions to stop TKI treatment. Patients’ concerns about cessation of TKI treatment and their psychological and emotional experiences during TFR are not adequately reported in clinical studies.

In clinical settings, the assessment of health-related quality of life (HRQoL) outcomes has gained significance in several aspects of patient care, including an association with key related outcomes, therapy failure, mortality and additionally as a primary end-point in oncology clinical trials (27–32). HRQoL is an inherently dynamic, multi-dimensional, and integrative index comprising no single best tool but rather different methods to measure a particular condition (33, 34). Different data collection methods might provide altered conclusions about a patient’s trial experience and the impact of their trial involvement on quality of life. When assessing patients enrolled in phase 1/2 oncology trials, the outcomes obtained from questionnaires showed no changes over time while evaluation *via* in-depth interviews described the patient’s perspective on psychological, emotional, and social impact derived from their participation in a clinical trial (35). Similarly, more studies have employed only quantitative tools for quality of life assessment with less emphasis on the usage of qualitative methodology that would better support the understanding of patient experiences (36–41). Therefore, to justify a rationale for any intervention and treatment approach, a combination of HRQoL assessment and objective clinical indicators ought to consider in study design and implementation process to better evaluate the treatment effectiveness and appropriateness through high-quality data collection and interpretation (40).

The present study was designed as a psychological substudy of ENESTPath (the Italian CML patients’ voice study) that would better assess the emotional experience of patients with CML-CP in Italy during and after stopping nilotinib therapy. The purpose was to get a deep and wide comprehension of the psychological status, not limited to the more assessed psychological outcomes as anxiety, depression, or quality of life, but also exploring the emotional experience starting from the own individual patient’s perspective. To do that, the CML patients’ voice study is designed as a mixed method study that uses both quantitative and qualitative approaches to explore patients’ psychological outcomes, health-related quality of life, and their experiences of being involved in the ENESTPath trial and discontinuation of nilotinib, with a particular focus on the time when patients completed the consolidation phase and approached the TFR phase.

The objectives of this substudy include assessment of personality traits, psychological distress, the strategies that patients adopt for coping during the consolidation phase of the ENESTPath trial and HRQoL from the consolidation to the TFR phase. Additionally, the emotional experience of patients who undergo treatment discontinuation during the TFR phase, and while in TFR phase and need to resume therapy due to relapse will be explored. The emotional experience of patients who are not eligible for randomization and have to withdraw from the substudy will also be explored.

## Methods

### Study Design

**Figure 1:**
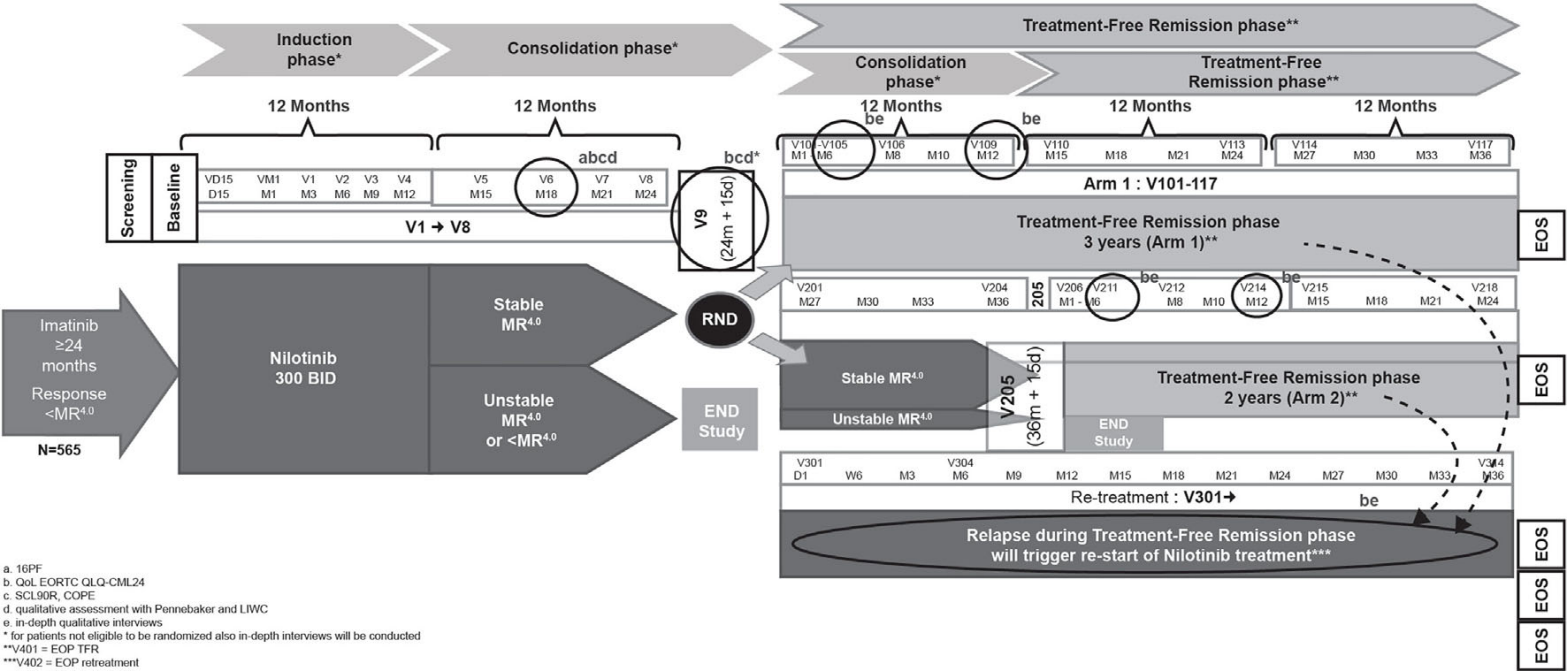
ENESTpath Italian sub-study design.

The Italian CML patients’ voice substudy of the ENESTPath phase 3, multicenter trial will utilize a mixed method design (qualitative–quantitative) to evaluate the psychological and emotional characteristics of patients with CML. Patients enrolled in the ENESTPath study received 12 months of nilotinib 300 mg BID in the induction phase followed by a consolidation with nilotinib for further 12 months. The patients who had a sustained DMR (≥ MR^4^ in 4 of 5 PCR assessments, including the last assessment in the last 12 months) at the end of the first 24 months of treatment were randomized either to immediate discontinuation (arm 1: a total of 24 months of nilotinib treatment) or to a further consolidation of 12 months duration before entering TFR (arm 2: a total of 36 months of nilotinib treatment) (**Figure 1**).

The ENESTPath study design has been described previously (42). Briefly, all patients who were ineligible for randomization, including patients not in sustained DMR at 24 months of nilotinib treatment discontinued study treatment and received treatment at the discretion of the investigator. These patients were followed until death or for 5 years from baseline visit for survival. Patients who relapsed during the TFR phase (loss of MMR, or three consecutive confirmed losses of MR^4^) had to enter the re-treatment phase with nilotinib and subsequently remain on study until study period of 5 years. The mixed-method design of the substudy aims to maximize the type and variety of information collected and support deep understanding of the patient’s perspective.

### Study Population

Patients in the ENESTPath study who agreed to participate in the Italian CML patients’ voice study and signed the informed consent form were enrolled. Patients who consented to participate in the substudy had a confirmed diagnosis of Ph+ CML-CP and had been treated with front-line imatinib for a minimum of 2 years with complete cytogenetic response.

### Design and Time Frames

Month 18 of consolidation phase was chosen as the **“**emotional-baseline**”** for study patients as enough time will have passed since the disruption of switching treatments at study entry, but some time remains until potential entry into the TFR phase. Additionally, the randomization visit represents a crucial moment from an emotional point of view due to the uncertainty about treatment discontinuation and entrance into the TFR phase. Owing to these factors, the psycho-emotional outcomes and quality of life assessment will be done at month 18 and at randomization (24 months for both arms) before the patients are informed about their eligibility to continue the study in TFR phase. Quantitative evaluation of quality of life through EORTC and qualitative assessment *via* in-depth interviews conducted at month 6 and 12 of TFR phase. These two timepoints in the TFR phase will ensure inclusion of both patients who enter TFR and then relapse and patients who maintain a stable TFR. A qualitative assessment using in-depth qualitative interviews are conducted if patients relapse during the consolidation or TFR phase (within maximum one month from relapse) to explore their emotional experience as subjective response to interviews regarding re-initiation of nilotinib treatment.

### Assessments

The outcomes of psychological and HRQoL assessments, along with the emotional experiences of being involved in the ENESTPath trial and cessation of treatment for remission will be explored in different ways depending on the stage of the clinical trial. During the consolidation phase, the psychological assessment will be performed through quantitative tools that focus on personality, psychological distress and coping strategies, while emotional experiences during this phase will be collected through a qualitative tool (diaries).

During the TFR phase, relapsing TFR or end of phase (EOP), emotional experiences will be explored through a qualitative approach using in-depth, unstructured interviews. HRQoL will be assessed through the whole patient journey at different time points during both the consolidation and TFR phases (**Figure 1**).

### Outcomes and Tools

#### Quantitative Tools

Socio-demographic data including patient characteristics such as gender, age, education, marital status, and occupation will be collected in order to control potential confounders or to identify mediating factors of psychological outcomes. Occupation is considered an indirect index of the general income level, that is not collected due to strict ethical requirements in conducting a research that is founded by a pharma agency.

Personality assessment will be conducted through the 16 Personality Factor (16PF) questionnaire (43, 44) in its Italian version (16PF-5) (45–47). The 16PF-5 is a multiple-choice questionnaire with a comprehensive normal-range measurement of anxiety, emotional stability and behavioral problem. This method has been effective particularly in clinical trials where in-depth assessment of the patient is required. The questionnaire organizes personality traits into five global scores including extraversion, independence, tough-mindedness, self-control, and anxiety. As the tool explores the psychological characteristics related to personality that are typically stable and unchanging, the questionnaire will be deployed once at 18-month consolidation phase.

Patients’ psychological distress will be measured at month 18 of the consolidation phase and at randomization at month 24 using the Symptom Checklist 90 – Revised (SCL 90-R) questionnaire in its validated Italian version (48–50). The SCL-90-R is a multi-dimensional, 90-item questionnaire with a 5-point Likert scale ranging from 0 as ‘no problem’ to 4 as ‘very serious’. The self-report questionnaire evaluates various psychological problems and symptoms of psychopathology clustered in nine primary scales including somatization, obsessive-compulsive, interpersonal sensitivity, depression, anxiety, hostility, phobic anxiety, paranoid ideation and psychoticism. The questionnaire also includes three global indices including Global Severity Index (GSI), Positive Symptom Distress Index (PSDI), Positive Symptom Total (PST), i.e., number of self-reported symptoms. The GSI, which encompasses a broad range of symptoms of psychopathology, will be used as an index of overall psychological distress. Higher GSI scores indicate greater distress and the clinical cut-off is above 55 T-score (or 1.0 raw score, 48-50).

Patients’ coping strategies will be assessed at month 18 of the consolidation phase and at month 24 at randomization through the Italian version of the Coping Orientation to Problems Experienced (COPE-NVI) questionnaire (51, 52). The questionnaire explores five dimensions of coping strategies including problem-orientation, avoidance, social-support, positive attitude and transcendent-orientation. With respect to the COPE scale, the higher the score, the more the coping strategy is used. Generally, avoidance strategies are dysfunctional, while social-support, positive attitude and transcendent-oriented strategies are functional and protective strategies.

HRQoL specific for patients with CML will be measured at month 18 during the consolidation phase, at month 24 at randomization and at months 6 and 12 of the TFR phase through the European Organization for Research and Treatment of Cancer adapted for patients with CML (EORTC QLQ-CML24) (53). The questionnaire consists of a 30-item general section (EORTC QLQ C30) plus 24 items specifically designed for patients with CML. The EORTC QLQ C30 assesses the following dimensions: global health status, physical functioning, role functioning, emotional functioning, cognitive functioning, and social functioning. Higher scores indicate a better outcome in the HRQoL domain. EORTC QLQ-CML24 assesses the aspects of symptom burden, impact on daily life and on worry/mood, body image problems and satisfaction with care and social life. Higher scores indicate a worse outcome in the HRQoL domain, except for the two scales on satisfaction in which higher scores indicate a better HRQoL.

#### Qualitative Tools

The evaluation of inner emotional experiences during different phases of the clinical trial will be explored through differing qualitative methodologies: expressive writing during the consolidation phase and in-depth, qualitative interviews during the TFR phase or following relapse.

**Figure 2:**
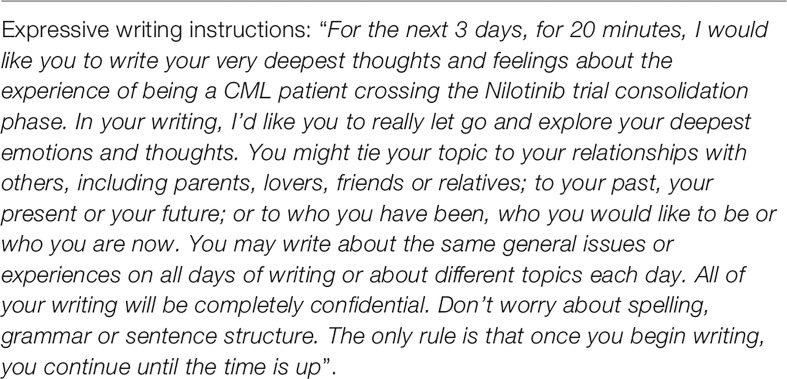
Writing instructions for diaries compilation at month 18 and at month 24.

Emotional experiences at month 18 during consolidation phase and at month 24 at randomization will be explored through Pennebaker’s method of expressive writing (54). The standard expressive writing paradigm was followed (55), with writing instructions as follows: (**Figure 2**)

This methodology allows an in-depth exploration of the cognitive and emotional thoughts relating to a particular experience and it is often used in different contexts (such as heart failure, HIV, psoriasis, mood and sleep disorders) both from a therapeutic or a research perspective. This technique has the advantage of a qualitative methodology with the strength of a quantitative tool, because the patients’ narrative might be analyzed both in terms of contents/interpretative level and through a quantitative coding. The quantitative coding and its data extraction will be performed through the Linguistic Inquiry and Word Count (LIWC) software (56) following a standard expressive writing paradigm (55, 57). The emotional experiences of patients being ineligible for randomization, and/or either achieving or failing TFR will be explored using in-depth, unstructured qualitative interviews according to the interpretative phenomenological analysis (IPA). Interviews will be guided using open-ended questions and will follow the narrative given by the patients to the facilitating interviewer. The patients will describe the disease course and their involvement in the TFR trial, including their experiences during discontinuation of treatment with TKI. With respect to the criteria “being TFR patients”, the interviews will be guided using open-ended questions and the emotional experience of being in the TFR phase and discontinuing nilotinib treatment will be explored. At the beginning of the interview, patients were asked to describe the emotional experience of this particular moment (example, “what is your emotional experience as a CML patient during the TFR phase?”). The interviews will follow the patients’ narrative flow and as the story unfolds, possible additional questions were 1) What are the critical aspects of your experience in general?; 2)What are the critical aspects of your experience linked to the healthcare setting?; 3) Thinking to your experience, which kind of resources are useful to face this particular phase (i.e. TFR)? A similar approach will be used for patients either failing to achieve TFR and being not eligible for randomization and the content of the interview questions will be decided accordingly. All interviews will be conducted by a clinical psychology researcher, audio recorded, transcribed verbatim and will be analyzed as per the principles of IPA (58–60).

### Data Analysis Plan

Quantitative data will be analyzed using SPSS Statistic Software. T-tests will be performed to test the changes in psychological distress, coping strategies and HRQoL from consolidation to randomization visits. Repeated measures of analysis of variance (ANOVA) will be performed to assess the stability or the changes in quality of life outcomes (EORTC QLQ-CML24) throughout the consolidation and TFR phases. Pearson’s correlation analyses will be performed to evaluate the relationship between psychological distress or HRQoL outcomes with coping strategies and personality traits. If the power of the sample is adequate, multivariate ANOVA will be employed to evaluate the relationship between the psychological and quality of life outcomes and the personality profile (16PF-5 factors) to clarify whether some personality characteristics might mediate or moderate psychological wellbeing of patients with CML during the consolidation and TFR phases.

Diaries will be analyzed using LIWC software. LIWC is an efficient method for measuring emotional, cognitive, structural and process components present in individuals’ verbal and written speech samples. The LIWC 2007 applications are designed to analyze the written text on a word by word basis, calculate the percentage of words in the text that match each of up to 82 language dimensions, and generate output as a tab-delimited text file that can be directly read into SPSS to construct thematic areas.

The verbatim records of the interviews will be analyzed through the use of principles of IPA (58, 61), a widely employed qualitative research approach in health psychology. This method allows to explore the individuals’ live experiences and by means they attribute sense to the experiences. Through a deep interpretation of individual narratives, IPA might allow the researcher to grasp the essence of how patient experience and understand the world (62, 63). This research approach is specifically followed to gain an understanding on subjective processes through which patients (64–66) will make sense of their symptoms or diseases. The process of IPA aids to uncover inherent beliefs and emotions that are carried in the stories by the deep analysis of their verbal statements, although writers are not necessarily completely cognitively aware of these psychological states (67). The analysis proceeds as a spiral from details to the whole, from one theme to the complexities of theme, from narratives to concepts and again to narratives, i.e., IPA is an iterative inductive process that starts from the detailed reading of the texts aimed at gaining a holistic understanding of all the collected narratives. The analysis proceeds step-by-step, in order to detect the main analytic themes of the texts and their interconnections (60). Core themes are generated in two ways: (1) by clustering similar themes of the various accounts into more exhaustive categories and (2) by formulating an interpretative hypothesis based on issues that might be best interpreted collectively. Finally, excerpts of the accounts are selected to exemplify the results.

#### Ethics

The study has been carried out in accordance with Good Clinical Practice guideline. As the CML patients’ voice study is a multicenter trial involving many hospital centers in Italy (Bari, Bologna, Cona, Livorno, Milano [two hospitals], Mirano, Orbassano, Pagani, Parma, Perugia, Pescara, Pisa, Reggio Emilia, Roma [two hospitals], Taranto, Verona, Vicenza), the research protocol has been approved by the Italian Health Authorities, by the Ethics Committee of the coordinator site (AO Università di Bologna Policlinico S.Orsola - Malpighi, Bologna, Italy) and by the Ethics Committees of all the hospitals involved. All patients gave written informed consent in accordance with the Declaration of Helsinki.

## Anticipated Outcomes

The study is planned to enroll at least 60 patients in the consolidation phase. At least 30 patients (10 not eligible for randomization, 10 in TFR and 10 failing TFR) will be planned to undergo qualitative interviews.

The study is planned to explore whether patients with CML have some common personality characteristics. With respect to the quantitative data, the study outcomes might show: (1) increased psychological distress when approaching randomization; (2) improved HRQoL with nilotinib treatment; (3) dysfunctional coping strategies (detachments) and personality traits (anxiety and tough-mindedness) correlated with psychological distress and HRQoL impairments. With respect to TFR, the study outcomes might reveal a better HRQoL in TFR when compared to the consolidation or randomization timepoints. In particular, we would expect all the HRQoL subscales (global health status, physical functioning, role functioning, emotional functioning, cognitive functioning, and social functioning, symptom burden, impact on daily life and on worry/mood, body image problems and satisfaction with care and social life) to indicate significantly better HRQoL (that means higher scores in all the EORTC QLQ C30 subscales and in the two scales on satisfaction of EORTC-QLQ-CML24, while lower scores in the remaining EORTC-QLQ-CML24 subscales) in TFR when compared to both the consolidation and randomization timepoints. The association of psychological distress with specific coping strategies might be expected to modulate patients’ perceived HRQoL. Overall, the quantitative findings will be integrated and deepened through qualitative findings. Since qualitative data follow a bottom-up approach, no *a priori* hypothesis has been made. Furthermore, both diaries and interviews will be analyzed to explore the emotional experience of patients with CML from their own perspective in order to gain new insights on their experiences. A set of preliminary data regarding the emotional experience of patients who are not eligible for randomization and ought to withdraw from the substudy have already been analyzed and the findings have been published previously (42).

## Discussion

Clinical practice guidelines for CML have so far not included psycho-emotional issues related to stopping TKI treatment and attempting TFR (7, 26). Few studies have been conducted to investigate the emotional and psychological ramifications of involvement in CML TFR trials, although data relating to treatment efficacy, safety, and interruption are becoming increasingly significant. Recent studies have shown that young patients with heavy CML symptom burden and short disease duration are more likely to attempt TFR. These patients also expressed several perceived benefits beyond treatment cost associated with TFR, including relief of medication side effects and convenience (68). In another study, the majority of patients with DMR indicated their willingness to attempt TFR and sought information on potential risks and benefits of TKI discontinuation, the conduct of the TFR phase and the probability of maintaining remission without recurrence. The study also revealed the scope of concerns to be addressed through qualitative analysis of survey responses and the need to collect and assess additional information regarding TFR (69). Though studies have reported patients’ anxiety toward stopping therapy for disease recurrence, appropriate counselling measures considering their psycho-emotional issues are not documented in routine clinical practice.

The vast majority of published studies have assessed quality of life using quantitative methods, namely EORTC QLQ-CML24 (53, 68), FACT-LEU (70) or SF-36 (71) questionnaires, while the use of qualitative methods to explore the psycho-emotional perception prior to and following TFR is so far scarce although it can highlight new and unexpected aspects of the patients’ wellbeing/distress starting from the patient’s perspective, adopting a patient-centered model of care and research. Of note, limited sample size might potentially impact the quantitative assessment and analyses, and hence integration of the qualitative data (both with diaries and interviews) could provide important sources of information also in cases of very small number of involved patients (59).

Despite the appeal of TKI cessation, it might be quite challenging to attempt to evaluate from a patient’s perspective. TKI discontinuation and TFR hold great potential for the improved management of CML in the near future, primarily through assessing how and when patients consider treatment interruption. Correspondingly, the fear of relapse and resistance to re-initiation of TKI therapy following TFR needs further understanding.

The CML patients’ voice substudy is unique as it is designed for in-depth assessment of a wide range of possible psychological and emotional variables along with an inner understanding of the patient’s experience directly from his/her perspective and words, that might provide better clinical practice insights for informing future discussions with patients who are considering treatment termination. Furthermore, this study design might provide valuable insights to plan future trials on CML treatment discontinuation. Finally, the findings of the Italian sub-study might help the clinicians in understanding the patient’s emotional experience in facing drug changing or innovations. We might assume that the qualitative findings might help to better understand the quantitative outcomes (psychological distress, coping strategies, and quality of life), for example sheding light on how patients give meaning to drugs and to being involved in a clinical trial, that could be potentially strictly linked with one’s own individual disease experience. These insights could help clinicians in tailoring their communication and interaction with patients, having in mind the complexity of the patients’ emotional experience.

## Data Availability Statement

The original contributions presented in the study are included in the article/supplementary material. Further inquiries can be directed to the corresponding author.

## Author Contributions

All authors met ICMJE criteria and all those who fulfilled those criteria are listed as authors. All authors had provided direction and comments on the manuscript. All authors contributed to the article and approved the submitted version.

## Funding

The study was supported by Novartis Pharma AG in relation to the substudy Clinical Trial Protocol-CAMN107AIC05 “CML patients’ voice: A pilot study exploring the emotional experience of patients during the ENESTPath study and its discontinuation”.

## Conflict of Interest

LB received a research fellowship from her institution on the project titled “CML patients’ voice: A pilot study exploring the emotional experience of patients during CAMN107AIC05 study and its discontinuation” funded by Novartis Pharma AG. SSu and LC are employees of Oncology Region Europe, Novartis Farma SpA, Origgio, Italy. EV received grant support, paid to her institution, from Novartis for the research project titled "CML patients’ voice: A pilot study exploring the emotional experience of patients during CAMN107AIC05 study and its discontinuation". MC received honoraria from Novartis, Celgene and Janssen. MBo received honoraria (advisory board) from Novartis, Pfizer, Incyte and Amgen.

The remaining authors declare that the research was conducted in the absence of any commercial or financial relationships that could be construed as a potential conflict of interest.

The authors declare that this study received funding from Novartis Pharma AG. The funder had the following involvement in the study: study design, writing of the article, and decision to submit it for publication.
